# Survival Rates among Co-infected Patients with Human Immunodeficiency Virus/Tuberculosis in Tehran, Iran

**Published:** 2017-08

**Authors:** Ghodratollah ROSHANAEI, Masoud SABOURI GHANNAD, Jalal POOROLAJAL, Minoo MOHRAZ, Leila MOLAEIPOOR

**Affiliations:** 1. Modeling of Noncommunicable Diseases Research Center, Dept. of Biostatistics and Epidemiology, School of Public Health, Hamadan University of Medical Sciences, Hamadan, Iran; 2. Research Center for Molecular Medicine, Dept. of Microbiology, School of Medicine, Hamadan University of Medical Sciences, Hamadan, Iran; 3. Research Center for Health Sciences, Dept. of Epidemiology & Biostatistics, School of Public Health, Hamadan University of Medical Sciences, Hamadan, Iran; 4. Iranian Research Center for HIV/AIDS, Iranian Institute for Reduction of High-Risk Behaviors, Tehran University of Medical Sciences, Tehran, Iran; 5. Dept. of Epidemiology and Biostatistics, School of Public Health, Hamadan University of Medical Sciences, Hamadan, Iran

**Keywords:** Acquired immunodeficiency syndrome, Human immunodeficiency virus, Tuberculosis, Risk factors, Survival rate

## Abstract

**Background:**

The number of deaths related with co-infection of tuberculosis (TB) and HIV remains inappropriately high worldwide. TB is anticipated to be the major reason of HIV-related deaths globally. This study aimed to find out and evaluate the characteristics of the possible risk factors influencing the survival time of co-infected patients with HIV/TB in Tehran the capital of Iran.

**Methods:**

This retrospective study was performed on the referred patients to the one of two Behavioral Diseases Counseling Centers, Imam Khomeini, and Zamzam Centers, Tehran, Iran, in 2004–2013. Data were analyzed by Cox PH model utilizing SPSS16 statistical software.

**Results:**

Multivariate analysis confirmed that the age at diagnosis (P=0.014), gender (P=0.002), sexual transmission (P=0.01), cotrimoxazole preventive therapy (P<0.001), and onset to TB after post-HIV diagnosis (P=0.01) were the parameters which had significant effects on the death of HIV/TBco-infected patients.

**Conclusion:**

The results, recommend interplay between different risk factors and the risk of death in co-infected patients with HIV/TB. We presented the barriers to higher-level organizational and functional integration for commitment to interfere with the modifiable risk factors, which effect on the mortality of patients.

## Introduction

The number of deaths related with co-infection of tuberculosis (TB) and HIV remains inappropriately high worldwide (1). TB is anticipated to be the major reason of HIV-related deaths globally (2). In spite of this, in 2013, almost only a third of identified HIV-positive TB, patients were treated with antiretroviral therapy (ART) globally (3). Moreover, the unpredicted high death in HIV-TB co-infected patients, even in a population with high income who have the right to use good healthcare and ART, confirm the importance of improving active and latent TB cure in HIV-infected patients (4).

Despite the new findings regarding the risk factors influencing the death of HIV/TB co-infected patients, there are still barriers to achieve and address complete understanding of these factors in different health systems (1). This may help how best to have integrated management of TB/HIV services. On the other hand, harm reduction is a helpful reaction to HIV transmission and other harms clearly covered by the global fund to battle AIDS, TB, and Malaria (5). The predictable global requirement funding for harm declined in 2015 alone was US$ 2.3 billion, which seemed necessary. Therefore, an obvious global funding crisis for harm reduction remains as a vital sanitation problem (5).

Progressive activities have been made to assess many of the health and social effects influencing the increased rate of HIV in Iran (6). Iran has registered 24290 HIV-positive patients until April 2012 (7). The number of people living with TB was almost 10485 in Iran, including 326 (approximately 2.2%) HIV-infected patients (8). Death in HIV/TB co-infected patients may be hinder. In order to approach this goal, it is essential to specify the higher risk of death in this group of patients.

This study was a retrospective study, which aimed to find out and evaluate the characteristics of the possible risk factors influencing the survival time of co-infected patients with HIV/TB in Tehran the capital of Iran. These findings may help to understand and highlight the vital need to recover the prevention, diagnosis, and treatment of co-infection patients with HIV/TB to strengthen the HIV cure program in TB patients in this part of the world.

## Materials and Methods

This study performed for the referred patients to the one of two Behavioral Diseases Counseling Centers, Imam Khomeini, and Zamzam Centers, Tehran, Iran, during 2004–2013, Iran. The data of co-infected patients with HIV/TB was recorded regardless of age at disease diagnosis (year) and gender but aged equal to or greater than 18 yr. Further data on TB infection was obtained from the Center for Disease Control and Prevention. Demographic data including gender, age at disease diagnosis, marital status, and educational level, behavioral information (imprisonment, smoking and narcotic/alcohol abuse, were recorded in a designed questionnaire. The data was analyzed by Cox PH model utilizing SPSS16 statistical software and *P*-value was set at a significance level of 0.05. Analysis of univariate Cox model (unadjusted) showed significant variables, which further were analyzed in a multivariable Cox model. Since using Cox model is based on the proportional hazard assumption, in order to investigate the assumption, the Schoenfeld Residuals model was used.

In the current study, pulmonary TB was considered in patients with two positive sputum smears or one positive sputum smear plus a positive sputum culture or a chest X-ray. Moreover, sputum-negative pulmonary TB was considered in patients with two negative sputum smears but with a positive sputum culture or with signs and symptoms of pulmonary TB who did not respond to a two-week broad-spectrum antibiotic treatment. Extra-pulmonary TB was diagnosed in patients with positive TB culture and pathological evidence (caseous necrosis) (9). In addition, HIV-positive antibody in patients was confirmed by enzyme-linked immunosorbent assay that followed by a western blot test as well. AIDS stage was cleared by a CD4 count less than 350 cells/mm^3^ of blood in an HIV-infected patient (10).

## Results

**Fig. 1:**
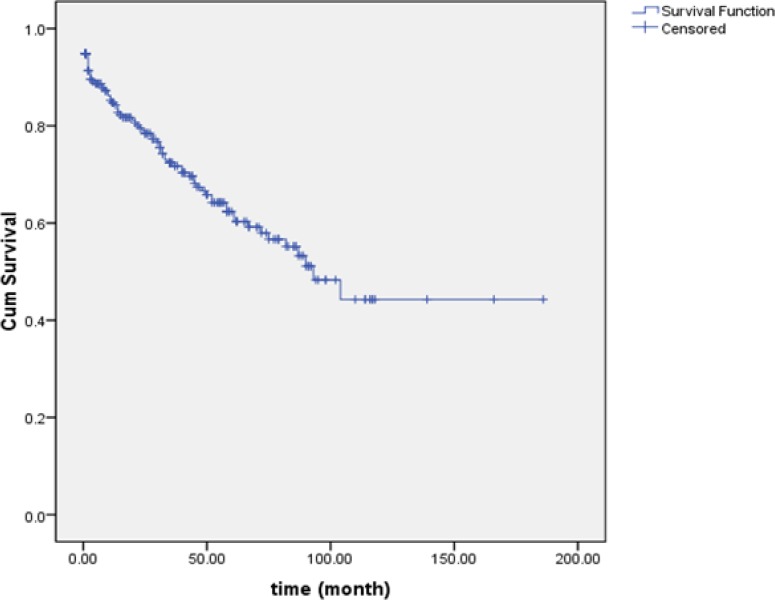
Survival probability of patients with HIV^+^/TB^+^

We identified 253 co-infected patients with HIV/TB included 232 (91.7%) male and 21 (8.3%) females. The mean (± SD) and median age at diagnosis for all patients were 36.6 ± 10.8 and 35 yr respectively. The mean (± SD) and median age of TB infection were 37.6 ± 11.1 and 36.1 yr. Period of infection onset to TB after HIV diagnosis was 32.1 ± 19 months (range, 1–122 months), respectively. Eighty patients (31.6%) died during the study. The mean (± SD) and median of survival time in the current research after diagnosis of the disease were 106.3 ± 7.3 and 93 months respectively in TB/HIV co-infected patients. In the current research, survival probability of patients with period of infection onset to TB after 3, 5 and 10 yr post diagnosis of HIV were 72%, 62.5%, and 44.3% respectively. HIV disease and co-infection to TB increased the rate of death in the patients (Fig. 1).

Table 1 shows the assessment of affected factors on the survival of HIV/TB co-infected patients by using of univariate Cox regression model. Analysis of univariate Cox model (unadjusted) showed that the variables including gender, stage of disease, sexual route of transmission, infection onset to TB after HIV diagnosis (yr), cotrimoxazole preventive therapy were significant (*P*<0.05) (Table 1). Table 1 shows variable gender significantly effect on the survivals of patients in the study so that the males were 4.88 times at risk of death in compare to females (*P*=0.027). Analysis of the disease stage showed that the risk of death in patients with HIV stage was 80% less than the patients in AIDS stage (*P*=0.024). Cotrimoxazole preventive therapy had significant effect on the survival of patients so that the patients without cotrimoxazole preventive therapy were 3.68 times at risk of death more than patients using cotrimoxazole (*P*<0.02). Analysis of sexual route of transmission showed that HIV-infected patients via spouse were 5.25 times more at risk of death than other modes of transmission (*P*=0.027). Patients with infection onset to TB after HIV diagnosis less or equal to 1 year were 2.2 times at risk of death than 3 or more than 3 yr period of HIV infection.

**Table 1:** The assessment of affected factor on survival of HIV^+^/TB^+^ patients by using of univariate Cox regression model

**Variables**	**Level**	**n (%)**	**Unadjusted OR**
Gender	Male	232 (91.7)	4.88(1.3–19)^*^
	Female	21 (8.3)	1
Age at time of disease diagnosis (yr)	<=35	127 (50.2)	0.8(0.5–1.09)
	>35	126 (48.8)	1
Marital status	Single	110 (45.1)	1.88(0.62–10.7)
	Married	78 (32)	1.74(0.52–5.8)
	Divorced	45 (18.4)	2.35 (0.9–8.3)
	Widowed	11 (4.5)	1
Educational level	Illiterate	18 (8)	1.53(0.6–6.3)
	Primary	71 (31.4)	1.96(0.8–3.86)
	Secondary	79 (35)	1.72(0.89–3.31)
	High & academic	58 (25.6)	1
Imprisonment	No	57 (22.5)	0.89(0.41–1.1)
	Yes	196 (77.5)	1
	No	73 (32.9)	0.81(0.49–1.35)
Smoking	Yes	149 (67.1)	1
Narcotic use	No	88(34.8)	0.85(0.53–1.12)
	Yes	165(65.2)	1
Alcohol use	No	232(93.5)	3.2(0.78–13)
	Yes	16(6.5)	1
Stage of disease	AIDS	183 (72.3)	1
	HIV	25 (9.9)	0.2(0.06–0.63) ^*^
	Unknown	45 (17.8)	0.58(0.23–1.4)
Cotrimoxazol preventive therapy	No	209(82.6)	3.68(1.7–8) ^*^
	Yes	44(17.4)	1
Isoniazid preventive therapy	No	(96.8)245	2.83(0.4–17.4)
	Yes	(3.2)8	1
Antiretroviral therapy	No	146(57.7)	1.1(0.67–1.7)
	Yes	107(42.3)	1
Sexual route of transmission	Homosexual - Heterosexual	75 (83.4)	1
	Spouse	15 (16.7)	5.25(1.5–21.8) ^*^
Route of HIV transmission	Intravenous drug users	116(46.4)	1
	Multiple exposure	87(34.8)	0.98(0.61–1.59)
	Sexual	22(8.8)	0.56(0.22–1.43)
	Other	28(10)	0.44(0.18–1.23)
Period of infection onset to TB after HIV diagnosis (year)	<= 1	107(56)	2.2(1.05–4.6)^*^
	2–3	42(22)	1.4(0.57–3.3)
	>3	42(22)	1

* Statistically significant

In the next step of data analysis, significant variables were analyzed in a multivariable Cox model. Since using of Cox model was based on the proportional hazard assumption, therefore in order to investigate the assumption, the Schoenfeld Residuals model was used. Goodness of fit statistical test based on the Schoenfeld Residuals showed that a Cox proportional hazard assumption was hypothesis valid (*P*=0.09). Therefore, all variables in the univariate and multivariate analysis that were *P*-value less than 0.2 included in the multivariate analysis (Table 2). Table 2 shows the age at diagnosis was significantly effective on the survivals of these patients so that the people aged less than 35 yr old were 0.41 times at risk of death in compare to those over 35 yr old (*P*=0.014). The effect of variable gender on the patient’s survival showed that men were at risk of death 6.7 times more than women were (*P*=0.02). Moreover, the risk of death in imprisonment patients was 1.1 times more than non-imprisonment, nevertheless, this variable had no significant effect on the patient’s survival (*P*=0.97). The risk of death in smokers was 2.29 times more than non-smokers, but with no significant effect on the survivals of drug users. The risk of death in people with no narcotic usage was reduced by 14%, but this variable had no significant effect on the patient’s survival (*P*=0.07).

**Table 2:** The assessment of affected factors on survival of patients with HIV^+^/TB^+^ by using multivariate Cox regression model

**Variables**	**Bivariate**	***P*-value**	**OR**	**OR-adjusted(95%CI)**	
				**L**	**U**
Age at time of disease diagnosis (yr)	<=35		1		
	>35	0.014	0.41*	0.2	0.83
Gender	Female		1		
	Male	0.002	6.7*	3.1	12.5*
Imprisonment	Yes		1		
	Yes	0.97	1.1	0.33	3.15
Smoking	No		1		
	Yes	0.146	2.29	0.750	6.970
Narcotic use	Yes		1		
	No	0.53	0.86	0.54	1.34
Route of HIV transmission	Other		1		
	IVD	0.161	1.75	0.8	3.79
	Multiple expose	0.27	2.72	0.46	16.1
	Sexual	0.01	9.8*	1.72	26.7
Cotrimoxazol preventive therapy	Yes		1		
	No	<0.001	8*	2.567	24.845
Stage of disease	AIDS		1		
	HIV	0.02	0.24*	0.07	0.8
	Unknown	0.14	0.68	0.1	1.39
Period of infection onset to TB after HIV diagnosis (year)	<= 1		1		
	2–3	0.11	0.54	0.25	1.16
	>3	0.01	0.29*	0.12	0.74
Marital status	Single		1		
	Married	0.47	1.336	0.62	2.91
	Divorced	0.79	1.133	0.46	2.82
	Widowed	0.54	1.683	0.32	8.82

* Statistically significant

Regarding route of HIV transmission, patients with intravenous drug abuse (IVD) and multiple expose transmissions were at risk of death 1.75 and 2.72 times respectively more than patients with unknown transmission route were.

However, the variables had no significant effect on the patient’s survival (*P* = 0.161 and *P*=0.27). However, in case of sexual transmission, the risk of death was almost 9.8 times more than people with unknown transmission, which had significant effect on the patient’s survival (*P*=0.01).

Cotrimoxazole preventive therapy had significant effect on the survival of patients so that the patients without preventive therapy were 8 times at risk of death more than patients using cotrimoxazole preventive therapy (*P*<0.001).

In view of stage of disease, the patients in the risk of death in patients with HIV stage was 76% less than the patients with AIDS stage which was statistically significant (*P*=0.02). In addition, the patients with unknown stage were at risk of death 32% less than patients with AIDS stage, but no significant statistical was reported (*P*=0.14).

Infection onset to TB after 3 yr post-HIV diagnosis had 71% less risk of mortality than patients with equal or less to period of 1 year TB infection (*P*=0.01).

## Discussion

The assessment of risk factors by univariate Cox model (unadjusted) showed that the variables including gender, stage of disease, sexual route of transmission, infection onset to TB after HIV diagnosis, cotrimoxazole preventive therapy were significant (*P*<0.05). Gender variable had significantly effect on the survivals of patients so that the people who were male were 4.88 times at risk of death in compared to females (*P* **=**0.027). This was inconsistent with our previous study in Hamadan, a western Province in Iran (11).

The analysis of data showed that the risk of death in patients with HIV stage was 80% lower than the patients in AIDS stage (*P* **=**0.024). The results seem logic because opportunistic infections and deaths are less common among HIV-infected patients (12) than people in AIDS stage.

Our results showed that cotrimoxazole preventive therapy had significant effect on the survival of patients so that the patients living without cotrimoxazole preventive therapy were 3.68 times at risk of mortality more than patients using cotrimoxazole preventive therapy (*P*<0.02). This is in accordance with a study in china (13) and is inconsistent with a research which reports the combination of isoniazid and antiretroviral therapy (*ART*) decreased the risk of TB or death by 65% in compare to ART alone (14). A possible explanation was confirmed by a review study that concluded evidence on the decreasing of all-cause mortality in consequence of early initiation of ART in TB/HIV co-infected Patients (15). Analysis of sexual route of transmission showed that HIV-infected patients via spouse were 5.25 times more at risk of mortality than other modes of transmission of infection (*P*=0.027). These findings are in consisting with our latest publication, which indicated that in co-infected patients with HIV/TB, married individuals were more susceptible to death than single people (11).

Patients with infection onset to TB after HIV diagnosis with less or equal to period of 1 year and 2–3 yr were 2.2 and 1.4 times respectively more at risk of mortality than the period of 3 yr HIV infection. There is still a gap in the data of the period of the highest mortality. However, from the observed outcome, period shortage of infection onset to TB after HIV diagnosis had a significant effect on the death in co-infected patients.

In the current study, variables with a *P*-value less than 0.2, which resulted from a univariate analysis, were entered into a multivariate Cox regression model to assess the influence of significant variables on the mortality of co-infected patients to HIV/TB (Table 2). Multivariate analysis also confirmed that the age at diagnosis (*P*=0.014), gender (*P*=0.002), sexual transmission (*P*=0.01), cotrimoxazole preventive therapy (*P*<0.001), onset to TB after post-HIV diagnosis (*P*=0.01) were the parameters which had significant effects on the death of HIV/TBco-infected patients. These results are inconsistent with another research in Tehran, which indicated that the AIDS-related mortality was related to gender, TB co-infection and antiretroviral therapy significantly (16).

Multivariate analysis found that the age at diagnosis was significantly effective on the survivals of these patients (Table 2), indicating that the risk of mortality diminished as 59% in the people aged less than 35 yr old, in compare to those who were over 35 (*P*=0.014) which may be due to the waning immunity in elderly people. These findings are similar to a recent research in china (13). Our observation of the effect of gender on the patient’s survival revealed that men were significantly at risk of death more than women were (*P*=0.02) which are accordance with a research in china (13). The reason may be associated with the total number of men in the current research who were 91.7% vs. 8.3% of women. Nevertheless, our understanding of HIV infection in women remains sparse, as are options for what women can use to diminish their risk of acquiring HIV (17).

The risk of death in the participants was with no narcotic usage reduced by 14%. However, this variable had no significant effect on survival. This is in agreement with the literature, which declared substance-usage decreased the health of HIV-infected drug users so that increased morbidity and mortality was observed in HIV-infected drug patients in compare with HIV-infected individuals who did not use drugs (18).

We identified that in case of sexual route, the risk of mortality was significantly more than people with unknown transmission in co-infected patients. Our result is not in agreement with the recent finding in china that showed an increased risk of death in HIV-infected people with a transmission route other than sexual transmission (13). The discrepancy of our results with the literature remains to be clarified.

Another vital point that came out of our research was the treatment outcome of patients with cotrimoxazole, which had a heightened intensity of clinical recovery in patients so that the patients without preventive therapy were 8 times at risk of death more than patients using cotrimoxazole preventive therapy. This finding has an essential role in policy and programmatic implications for sanitation authorities. Nevertheless, our study suffered from a limitation so that we did not have access to ART history among HIV/TB co-infected patients. Thus, the crucial question of when the patients initiated combined antiretroviral treatment (cART) in relation to TB treatment, and how cART could effect on the association with delaying of death in such patients remained unclear and complicated. A study in the USA indicated that the co-infected patients should be given antiretroviral drugs during treatment for active TB before the finishing point of TB therapy so that the rate of mortality was reduced by 56% with initiation of antiretroviral therapy in that study (19).

Multivariate analysis found that the risk of death was significantly lower (76%) in patients with HIV stage than the patients with AIDS stage. Opportunistic infections and deaths are more common in people living with AIDS stage than HIV-infected patients (12). In addition, the patients with unknown stage were at risk of death 32% less than patients with AIDS stage, but no statistical significance was reported.

The current research showed that the participants infected to TB after 3 yr post-HIV diagnosis had 71% less risk of mortality than patients with equal or less to period of 1-year TB infection. The reason remains the main challenge and requires further research. However, major obstacles such as poverty and illiteracy and their consequences may be the reasons. In addition, this study suffered from a limitation so that we did not have access to ART history among HIV/TB co-infected patients. Thus, the crucial question of when the patients initiated combined antiretroviral treatment (cART) in relation to TB treatment, and how cART could effect on the association with delaying of mortality in such patients remained unclear and complicated.

Another limitation was that the study covered only Tehran, the capital of Iran. Therefore, it cannot be the representative of the whole country. Further studies are needed to identify the significant reliable predictors as risk factors for co-infected patients with TB/HIV national wide. There is a need for better quality evidence around how best to deliver integrated services to strengthen the HIV treatment cascade in TB patients, both at primary healthcare level and within community settings. Obviously, the expected consequence of such strategies will diminish the proportion of mortality in HIV/TB co-infected patients.

## Conclusion

We specified in this study set of modifiable and non-modifiable high-risk factors affecting the mortality of patients co-infected with HIV/TB. The current research recommends interplay between various risk factors and the risk of death in co-infected patients with HIV/TB. We also presented the barriers to higher-level organizational and functional integration for commitment to interfere with the modifiable risk factors, which affect on the mortality of patients.

## Ethical considerations

Ethical issues (Including plagiarism, informed consent, misconduct, data fabrication and/or falsification, double publication and/or submission, redundancy, etc.) have been completely observed by the authors.
